# Relationship between Lipid Phenotypes, Overweight, Lipid Lowering Drug Response and *KIF6* and *HMG-CoA* Genotypes in a Subset of the Brisighella Heart Study Population

**DOI:** 10.3390/ijms19010049

**Published:** 2017-12-24

**Authors:** Sabrina Angelini, Martina Rosticci, Gianmichele Massimo, Muriel Musti, Gloria Ravegnini, Nicola Consolini, Giulia Sammarini, Sergio D’Addato, Elisabetta Rizzoli, Dauren Botbayev, Claudio Borghi, Giorgio Cantelli-Forti, Arrigo F. Cicero, Patrizia Hrelia

**Affiliations:** 1 Department of Pharmacy and Biotechnology, via Irnerio 48, University of Bologna, 40126 Bologna, Italy; gianmichele.massimo3@unibo.it (G.M.); gloria.ravegnini2@unibo.it (G.R.); nicola.consolini2@unibo.it (N.C.); giulia.sammarini2@unibo.it (G.S.); daur_92_05@bk.ru (D.B.); patrizia.hrelia@unibo.it (P.H.); 2 Department of Medical and Surgical, University of Bologna, 40126 Bologna, Italy; mrosticci@yahoo.com (M.R.); sergio.daddato@unibo.it (S.D.); elisabetta.rizzoli@unibo.it (E.R.); claudio.borghi@unibo.it (C.B.); arrigo.cicero@unibo.it (A.F.C.); 3 Department of Public Health, Epidemiological Service, Local Health Authority of Bologna, 40126 Bologna, Italy; m.musti@ausl.bologna.it; 4 Department of Biotechnology, Faculty of Biology and Biotechnology, Кazakh National University Named after al-Farabi, 050040 Almaty, Kazakhstan; 5 Department for Life Quality Studies, Corso d’Augusto 237, University of Bologna, 47921 Rimini, Italy; giorgio.cantelliforti@unibo.it

**Keywords:** *HMG-CoA*, *KIF6*, polymorphisms, hypercholesterolemia, waist circumference, Brisighella heart study

## Abstract

The existence of genetic traits might explain the susceptibility to develop hypercholesterolemia and the inter-individual differences in statin response. This study was performed to evaluate whether individuals’ polymorphisms in *HMG-CoA* and *KIF6* genes are independently associated with hypercholesterolemia, other lipid-associated traits, and statin response in unselected individuals enrolled in the Brisighella heart study (Survey 2012). A total of 1622 individuals, of which 183 under statin medication, were genotyped for a total of five polymorphisms (KIF6 rs20455, rs9471077, rs9462535; HMG-CoA rs3761740, rs3846662). The relationships between the five loci and clinical characteristics were analyzed. The principal basic parameters calculated on 12 h fasting blood included total cholesterol (TC), High Density Lipoprotein Cholesterol (HDL-C), Low-Density Lipoprotein Cholesterol (LDL-C), and triglycerides (TG). Hypercholesterolemia was defined as a TC >200 mg/dL or use of lipid-lowering medication. 965 individuals were characterized by hypercholesterolemia; these subjects were significantly older (*p* < 0.001), with body mass index (BMI) and waist circumference significantly higher (*p* < 0.001) compared to the others. *HMG-CoA* rs3846662 GG genotype was significantly over-represented in the hypercholesterolemic group (*p* = 0.030). *HMG-CoA* rs3846662 genotype was associated with the level of TC and LDL-C. Furthermore, in the same subset of untreated subjects, we observed a significant correlation between the *KIF6* rs20455 and HDL-C. *KIF6* variants were associated with a significantly lower (rs20455) or higher (rs9471077 and rs9462535) risk of obesity, in males only. No association between responsiveness to statins and the polymorphisms under investigation were observed. Our results showed associations between *HMG-CoA* rs3846662 and *KIF6* rs20455 and lipid phenotypes, which may have an influence on dyslipidemia-related events. Moreover, this represents the first study implicating *KIF6* variants with obesity in men, and point to the possible involvement of this genetic locus in the known gender-related differences in coronary artery disease.

## 1. Introduction

Cardiovascular diseases represent the leading causes of mortality and morbidity worldwide and contribute remarkably to the increase of health care expenditures [1]. Based on these considerations, addressing cardiovascular diseases requires concrete actions and, in particular, a strategy, both globally and nationally, which includes prevention and reduction of risk factors, and adequate surveillance and monitoring [2]. Epidemiological studies have established the benefit of reducing low-density lipoprotein cholesterol (LDL-C) and triglycerides (TG) in diminishing cardiovascular disease events [2,3,4,5,6]. In light of these studies, the use of lipid-lowering drugs, mainly statins, may represent a primary prevention strategy of excellence. The significance of this approach is supported by a recent work by McConnachie et al. showing that a five-year primary prevention treatment with statins leads to long term benefits, reducing coronary-causes of death, and diminishing first hospital admission for cardiovascular events [7]. Statins are the first choice LDL-lowering drugs with a recognized universal efficacy in preventing vascular events. The recognition of statin efficacy is highlighted by a recent suggestion to expand the use of statins even in individuals with a low-risk profile [6,7]. Despite the enthusiasm, we have to pay attention to some uncertainty related to the non-achievement of the desired LDL-C targets in about 50% of the high-risk treated population, and the occurrence of cardiovascular events even in patients who achieve the LDL-C levels [8,9,10]. These two aspects suggest the existence of genetic traits that might explain the susceptibility to develop hypercholesterolemia and, secondly, the inter-individual response under statin regimen.

Several genome-wide studies have highlighted the involvement of numerous genes in lipid metabolism, in particular controlling serum High Density Lipoprotein Cholesterol (HDL-C), LDL-C and TG [11,12]. These loci include common variants, which have been consistently associated with lipid levels in candidate-gene studies reported over the past decade. Currently, the most studied variants are the G to A base change affecting the splicing site of exon 13 in the 3-hydroxy-3-methylglutaryl coenzyme A (*HMG-CoA*) reductase (rs3846662) and the arginine to tryptophan substitution at codon 719 in the Kinesin-like protein 6 gene (*KIF6*, rs20455). HMG-CoA reductase is the rate-limiting enzyme in cholesterol biosynthesis and the LDL-C-lowering effect of statins are mediated through its inhibition [13]. KIF6 belongs to a superfamily of motor proteins (kinesins) that act upon microtubules as intracellular transport of cargo, including membrane organelles, protein complexes and mRNAs within cells [14]. KIF6 is not directly involved in lipid metabolism; however, several studies reported the association between *KIF6* gene polymorphisms, coronary heart disease and events reduction from statin therapy [15,16,17,18,19,20,21]. All these considerations prompted us to perform a pharmacogenetic study to determine whether individuals’ polymorphisms in *HMG-CoA* reductase and *KIF6* genes are independently associated with hypercholesterolemia, other lipid-associated traits and statin response in a sample of unselected individuals enrolled in the Brisighella Heart Study (BHeS). Given the complexity of the investigated traits, we selected other polymorphisms, in addition to the two previously described, already associated with serum lipids levels and/or cardiovascular events [19,22]. In the present study, we also explored association between the investigated polymorphisms and obesity. This last association is driven by the fact that obesity is among the cardiovascular risk traits, together with lipid levels. Moreover, obesity is a well-established contributor to the development of dyslipidemia [23]. 

## 2. Results

### 2.1. Study Population: Main Characteristics and Genotype Distribution

The cohort we studied included 1622 subjects participating to the 2012 BHeS survey; main demographic, clinical and laboratory parameters are reported in Table 1. A total of 965 individuals were characterized by hypercholesterolemia; the hypercholesterolemic group included individuals with TC >200 mg/dL (*n.* 782) or under statin therapy (*n.* 183). Hypercholesterolemic subjects were significantly older (*p <* 0.001), with body mass index (BMI) and waist circumference significantly higher (*p <* 0.001) compared to the non-hypercholesterolemic individuals. With regards to lipidic parameters, all of them (LDL-C, HDL-C, TG, apoB and apoA1) were significantly higher in subjects with hypercholesterolemia, compared to non-hypercholesterolemic individuals (*p <* 0.001, for all); even though there was a relatively wide standard deviation in both groups (Table 1).

Deviation from the HW equilibrium was observed for a single polymorphism (*KIF6*, variant rs9462535) in the group of subjects with no hypercholesterolemia; no departure from the HW equilibrium was observed for any other polymorphism, either in the overall population or in the hypercholesterolemic, or not, groups (Table 2). Moreover, there was no significant difference in age, gender, BMI, waist circumference and clinical variables among genotype groups within the same group of study subjects. In the overall population, the minor allele frequency (MAF) is consistent with the one reported for a population from Toscana, Italy, and available on the 1000 Genome Project (http://www.1000genomes.org; Table S1).

### 2.2. KIF6 and HMG-CoA Genotypes and Lipid Phenotypes -TC, LDL-C, HDL-C, TG Levels

**Figure 1:**
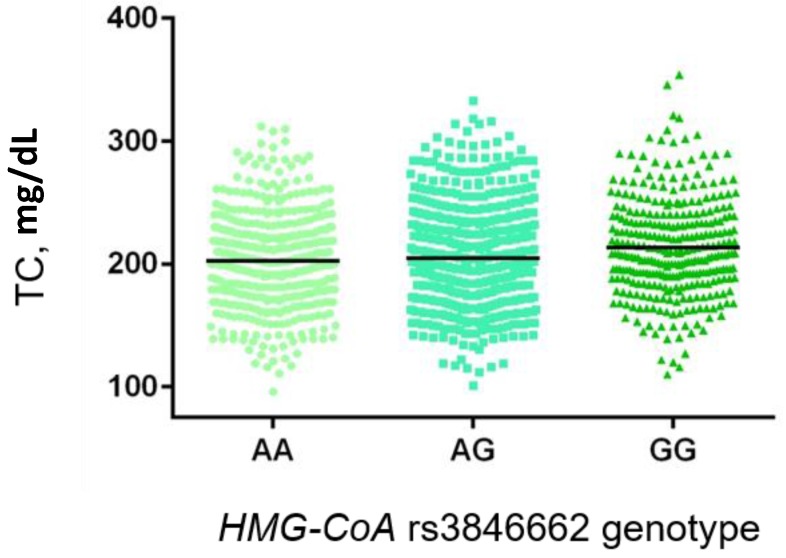
Association of the functional variant in *HMG-CoA* gene (rs3846662) with TC level in a subset of the Brisighella Heart Study population. Black lines represent TC mean value.

**Figure 2:**
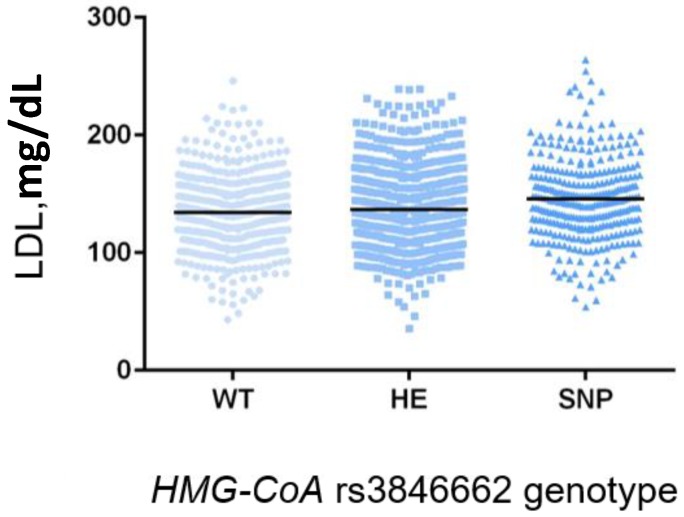
Association of the functional variant in *HMG-CoA* gene (rs3846662) with LDL-C level in a subset of the Brisighella Heart Study population. Black lines represent LDL-C mean value.

**Figure 3:**
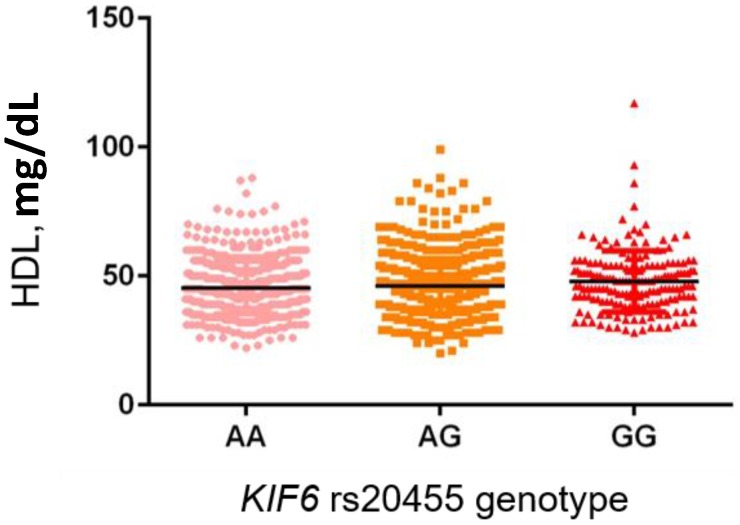
Association of the variant in *KIF6* gene (rs20455) with HDL-C level in a subset of the Brisighella heart Study population. Black lines represent HDL-C mean value.

Genotype distribution revealed a significantly different distribution of the *HMG-CoA* rs3846662 between hypercholesterolemic and individuals with normal TC value (Table 3). In particular, the GG genotype was significantly over-represented in the hypercholesterolemic group (23.6% vs. 18.7%, *p* = 0.030) with an OR 1.40 (95% CI 1.03–1.88, following adjustment for age, gender and BMI). None of the other analyzed polymorphisms showed association with hypercholesterolemia. Notably, excluding the subjects under lipid-lowering therapy (*n*. 183), the *HMG-CoA* rs3846662 genotype was also significantly associated with the level of TC and LDL-C (Figure 1 and Figure 2, respectively). In both cases the homozygous variant genotype (GG) was significantly associated with higher mean levels of TC and LDL-C compared to the AG and AA genotypes (TC: 212.9 ± 40.3 vs. 204.3 ± 38.8 and 202.6 ± 38.2, *p* = 0.003; LDL-C: 144.8 ± 35.2 vs. 136.5 ± 34.7 and 134.1 ± 33.0, *p* < 0.001). Furthermore, in the same subset of untreated individuals we observed a significant correlation between the *KIF6* rs20455 and HDL-C. In particular, carriers of the GG genotype showed a significantly higher level of HDL-C compared to the AG and AA genotypes (47.9 ± 11.9 vs. 46.2 ± 10.7 vs. 45.4 ± 10.4, Figure 3, *p* = 0.037). Interestingly, the wild-type (AA) genotype was also associated with higher level of LDL-C compared to the other genotypes (140.4 ± 36.1 vs. 135.7 ± 33.8 vs. 136.2 ± 31.9); however, the difference was only marginal (*p* = 0.085). None of the investigated polymorphisms were associated with TG levels.

### 2.3. KIF6 and HMG-CoA as Susceptibility Locus for Obesity Assessed as BMI and Waist Circumference

BMI was categorized using standard World Health Organization for healthy weight, overweight (≥25 and <30 kg/m^2^) and obese (≥30 kg/m^2^). Considering obesity as a well-established contributor to dyslipidemia development and cardiovascular risk traits, BMI was dichotomized in obese subjects (BMI ≥ 30.0 kg/m^2^) or not. Considering the overall population, stratified according to *KIF6* and *HMG-CoA* genotypes, none of the analyzed polymorphisms were associated with BMI (Table 4). We also considered the association with waist circumference that better assess body fat distribution compared to BMI. Waist circumference was first categorized according to the standard clinical guidelines for men (<94, 94–100, ≥100 cm) and women (<71, 71–90, ≥90 cm), corresponding to the BMI standard clinical categories for healthy, overweight and obese. The analysis was performed separately for men and women, dichotomizing the population in healthy weight (waist circumference <90 cm in women and <100 cm in men) or not. The analysis highlighted important gene-gender interactions. In particular, *KIF6* polymorphisms were associated with a lower (rs20455) or higher (rs9471077 and rs9462535) risk of obesity, in males only (Table 5). In particular, the rs2455 AG and GG genotypes were associated with lower risk of obesity (OR 0.61; 95% CI 0.43–0.86, *p* = 0.004, and OR 0.58; 95% CI 0.35–0.95, *p* = 0.029, respectively). On the other hand, rs9471077 -AA- and rs9462535 -CC- conferred risk for obesity (OR 1.72; 95% CI 1.06–2.79, *p* = 0.027, and OR 1.72; 95% CI 1.06–2.80, *p* = 0.027, respectively) following adjustment for age. No association or trend to was observed in females (Table 6).

### 2.4. KIF6 and HMG-CoA Genotypes and Statin Response

Among the studied subjects, approximately 60% (*n.* 965) suffered from hypercholesterolemia. Amongst them, only 19.0% (*n.* 183) were under statin therapy. To assess differential responsiveness to statins, we tested the association between the polymorphisms under investigation and LDL-C levels in treated individuals, considering as responsive the individuals with LDL-C <130 mg/dL. Neither the presence of *KIF6* or *HMG-CoA* polymorphisms was associated with treatment response (Table 7).

## 3. Discussion

Serum lipids are important determinants of cardiovascular events, strongly related to morbidity [11,24,25,26,27,28]. Therefore, screening for increased circulating lipid levels and early treatment with statins are a key strategy for cardiovascular event prevention in the clinical practice. Besides this, several studies have suggested that lipid phenotypes are linked to genetic factors. In view of this, many efforts have been made to identify potential lipid-associated loci that could contribute to increase the armamentarium of available preventive strategies. The identification of a genetic feature, associated with the lipid phenotype, has a pivotal role in stepping up targeted medical controls and allows prompt intervention. The lipid phenotype is certainly recognized as a complex trait, involving multiple genes, and with a significant gene-environment interaction [27]. In the attempt to overcome the genotype-phenotype gap in complex diseases, candidate gene/pathway approaches have traditionally been used. The success of these approaches strongly depends upon the correctness of the initial choice, which may lead to miss (i.e., unselected genes) significant results. In this context, genome-wide association studies (GWAS) represent a powerful tool. Starting from 2008, we witnessed an explosive rise in the number of GWAS identifying common genetic variants associated with LDL-C [11,12,28,29,30,31,32,33]. Among the discovered hits, three studies [12,32,33] reported the significant association of a variant in the *HMG-CoA* gene (rs3846662) and LDL-C level. The result was further replicated in gene-candidate studies [34,35,36]. Stimulated by all these studies, we replicated the finding on the *HMG-CoA* polymorphisms rs3846662, in an independent and homogeneous Italian population. Overall, all the studies agree on the association of the *HMG-CoA*-rs3846662-minor G allele with higher levels of LDL-C; in our study, in particular, the association was more evident in the homozygous GG genotype group. The polymorphism is a common variant located in intron 13 of the *HMG-CoA*, affecting alternative splicing of the exon. Indeed, an in vitro study by Burkhardt and colleagues highlighted its functional role in modulating alternative splicing of HMG-CoA mRNA in human lymphoblastoid cells homozygous for the *HMG-CoA* minor allele [37]. In particular, the variant allele is associated with a significantly lower mRNA level of the alternatively spliced (Δ13 exon) *HMG-CoA*. The authors speculated the polymorphism rs3846662 is located in a binding motif for a splice auxiliary protein, and allele status determines a change in the binding affinity of this protein. Consequently, a diminished HMG-CoA activity would promote a lower cellular cholesterol synthesis and, subsequently, an increase of cholesterol uptake from plasma through the LDL-receptor pathway to maintain intracellular cholesterol homeostasis [37,38]. The finding that the splice variant could not restore the enzyme activity when expressed in HMG-CoA deficient cells corroborates the functional role of the *HMG-CoA* polymorphism (rs3846662). Despite its pharmacological interest, the exact molecular mechanism governing this process is largely unknown. Most probably, the presence of the A-major allele in homozygous increases the proportion of HMG-CoA mRNA lacking exon 13 [37]. This alternative spliced variant is characterized by the lack of a portion of the catalytic domain (encoded by exon 13), and therefore may potentially affect stability of the enzyme [37,39,40]. Most recently, it has been recognized the existence of specific splicing factors influencing stability, translation and structure of mRNAs, contributing to the observed phenotypic trait [41]. In this context, the identification of HNRNPA1, a mRNA splicing regulator modulating the expression of HMG-CoA in an allele-related manner, and altering the RNA stability [40], takes on a great importance in pharmacology. Specifically, HNRNPA1 promotes *HMG-CoA* exon 13 skipping, more predominantly in the presence of the A-major allele, which also directly contributes to this exon-skipping phenomenon.

Besides the *HMG-CoA* polymorphism, we also observed a correlation between *KIF6* rs20455 and HDL level; in particular, homozygous carriers of the G-minor allele showed higher serum HDL-C. The rs20455 variant is a non-synonymous polymorphism, leading to the replacement of a non-polar Trp with a basic Arg at codon 719, widely screened in relation to cardiovascular events, serum lipids and statin treatment outcome in various populations, with contrasting results [20,21,42,43,44,45,46,47]. To the best of our knowledge, our study is the first reporting an association with the KIF6 719Arg variant and HDL-C, while no apparent difference or opposite results were observed in previous studies [20,21,42,43,44,45,46,47]. Interestingly, *KIF6* polymorphisms (rs20455, rs9471077 and 9462535) were associated with risk of obesity, calculated as waist circumference, but in males only. Currently, obesity represents one of the major challenges and a public health problem worldwide. Adiposity, in particular abdominal obesity, is recognized as the key contributor to diabetes and other chronic diseases, and as a risk factor for hypercholesterolemia and further cardiovascular risk traits [27,28,29]. Several studies have suggested that obesity is linked to genetic and environmental factors, and identifying genetic traits, including polymorphisms, may point to dysregulated genomic pathways. Recently, GWAS studies have identified several loci associated with obesity, mostly defined according to BMI values; however, none of them reported any association with *KIF6* variants [48,49,50,51,52]. In our study, we found no correlation between any of the analyzed genetic variants and BMI, while we identified an interesting gene-gender interaction assessing obesity through waist circumference. Appropriate measures to prevent traits associated with cardiovascular events (as diabetes, dyslipidemia, hypertension, etc.) are mostly taken according to BMI anthropometric measures, aimed at identifying obese individuals. However, in the last decade waist circumference has emerged as a more accurate predictor of the metabolic risk of obesity compared to BMI [53,54,55,56,57]. Based on these considerations, we analyzed men and women separately, as sex-specific cutoffs for waist circumference should be used to identify increased risk associated with abdominal fat. Our results showed that *KIF6* variants might confer risk of obesity in men only. This finding is interesting as the importance of gender in cardiovascular diseases has been widely described [58,59], and *KIF6* variants might potentially explain the gender-related differences in atherosclerosis development. *KIF6* encodes an intracellular protein involved in cellular cargo transportation, also expressed in coronary endothelial cells [60], intuitively pointing to a possible role in cardiovascular function. Diverse variants within this gene may differently alter intracellular transport in endothelial cells in men and women, predisposing to coronary endothelial dysfunction and coronary heart disease. Recently, a study by Yoshino and co-workers reported a significant association between *KIF6* rs20456 and coronary epicardial endothelial dysfunction in men only [61], while, in contrast, a previous study showed association between *KIF6* rs20455 and increased risk of coronary heart disease and myocardial infarction in Han Chinese women [43]. Overall, to establish with certainty if our gender-specific finding reflects true-causal association, rather than a chance result, further studies deepening the underlying mechanism are required. Indeed, the homogeneous population and uniformity of life-style, minimizing population bias, points to a possible genetic locus for obesity in men. However, the association between *KIF6* polymorphisms and obesity needs confirmation or refutation in additional studies.

With regard to lipid-lowering therapy with statins, we did not find any association between *HMG-CoA* or *KIF6* genotypes and achievement of <130 mg/dL LDL-C level. The data on KIF6 are in disagreement with the literature, as several studies have shown that *KIF6* 719Arg allele carriers might have a greater lipid-lowering response from statin therapy compared to non-carriers [15,16,17,44,62,63,64]. Furthermore, some (but not all) KIF6 719Arg carriers undergoing statin therapy have shown a greater reduction of coronary heart disease compared to non-carriers [17,18]. Although these findings have been debated, a *KIF6* (rs20455) genetic test is commercially available to physicians and could be routinely included in the clinical practice. A recent study has highlighted that provision of *KIF6*-testing results and supporting information directly to patients was associated with significantly higher adherence and persistence to statin therapy over six months of follow-up [46]. In the era of personalized medicine, this study suggests that pharmacogenetic testing approaches may represent a promising tool in the promotion of healthy patient behaviors, which is then translated into clinical utility (i.e., higher statin adherence and persistence). A different reason might be why we have not observed any association between *KIF6* genotypes and response to statin therapy. In particular, important limitations are: (i) sample size; (ii) statin responsiveness definition; (iii) kind of administered statin. With regard to the sample size, only 183 subjects out of 965 hypercholesterolemics were under statin therapy, greatly reducing the statistical power. However, this distribution is representative of treated subjects in the whole BHeS group where the decision to treat hypercholesterolemia is left to local General Physicians. Moreover, some cases of hypercholesterolemia were firstly diagnosed at the time of this population survey. Then, statin treatment is not considered as a first line for hypercholesterolemia in subjects at low global cardiovascular disease risk [65]. Regarding responsiveness, we used achievement of LDL-C <130 mg/dL as a measure of response, while a more appropriate analysis would be to consider percentage reduction from baseline. However, the study started in 1972, and participants were evaluated every 4 years, so that a reliable baseline level is impossible to establish. An alternative analysis would be considering percentage reduction from the previous BheS survey. However, this approach is not properly correct, as in a four-year interval, it is difficult to establish the adherence to statin therapy and, therefore, the lipid levels observed. Alternatively, we could measure participants’ lipid levels 6 months prior to the BheS follow-up; however, this option was not scheduled, therefore we cannot calculate percentage reduction. Lastly, concerning the issue of the kind of administered statins, to date, benefit of statin therapy for *KIF6* carriers has been demonstrated for atorvastatin and pravastatin treatment only. In our study, it is difficult to draw a pharmacogenetic conclusion, as subjects under lipid-lowering treatment are heterogeneous in terms of the type of statin and dosage.

In conclusion, our results showed important associations between *HMG-CoA* rs3846662 and *KIF6* rs20455 and lipid phenotypes, which may have an influence on dyslipidemia-related events. Next to this, to the best of our knowledge, this represents the first study implicating *KIF6* variants with obesity in men only, and point to the possible involvement of this genetic locus in the known gender-related differences in coronary artery disease risk.

## 4. Methods

### 4.1. Study Population

The BHeS is a prospective population-based epidemiological investigation, started in 1972, involving 2939 unselected individuals, free of cardiovascular disease at enrollment, all resident in the rural town of Brisighella, located in northern Italy [66,67,68]. Brisighella was selected as the typical example of a rural community in the process of industrialization, with a very low rate of immigration and/or emigration, that basically guarantees a good degree of genetic homogeneity, and above all with uniformity of lifestyle habits. Participants were clinically evaluated at baseline and every 4 years following enrollment, when extensive clinical and laboratory data were obtained. The BHeS protocol and its sub-studies have been approved by the Ethical Board of the University of Bologna (protocol n BHS72—follow-up, 17 April 2012). Study participation was on a voluntary basis; a formal consent was obtained and signed by all participants before inclusion for study participation and anonymous data publication in accordance with national legislation. Study participation could be withdrawn by any subject, at any time during the study, according to the Helsinki Declaration and later Amendments.

### 4.2. Phenotyping

This population sample, participating in the 2012 survey, included 1622 individuals, of whom 183 were under statin treatment. Main demographic, clinical, lipid and lipoprotein characteristics of the studied cohort are summarized in Table 1. The basic parameters were evaluated with standardized methods by trained lab technicians at the S. Orsola-Malphighi Hosptial, Bologna, in our non-accredited research lab on 12 h fasting blood included TC, HDL-C, total triglycerides (TG), apoB and apoA1. LDL-C was estimated with Friedewald formula. When TG values were higher than 400 mg/dL, the LDL-C values was measured by beta-quantification. 

Hypercholesterolemia was defined as a TC >200 mg/dL, or use of lipid lowering medications. We selected the value of 200 mg/dL based on the definition given by the National Center for Disease Prevention and Health Promotion. The criteria to start statin therapies were those suggested by the more recent European Atherosclerosis Society at the time the treatment began [69]. All the individuals included in the study were in primary prevention for cardiovascular disease. Subjects with high probability to be affected by heterozygous Familial Hypercholesterolemia (hFH) have been identified by the application of the Dutch Lipid Score and then a priori excluded from the analysis [70].

### 4.3. Genetic Analysis

Genomic DNA was isolated from EDTA-anticoagulated whole blood using the QIAamp DNA Blood kit (Qiagen, Hilden, Germany) as recommended by manufacturer. For genotype analysis, DNA samples were diluted and stored at −20 °C as 10 ng/μL aliquots. For DNA analysis, a total of 5 polymorphisms, 2 in the *HMG-CoA* reductase gene and 3 in the *KIF6* gene, were genotyped (Table S1). Genotyping was performed by RT-PCR using the 5′-nuclease allelic discrimination assay (TaqMan^®^, Applied Biosystems, Foster City, CA, USA), according to the manufacturer instructions. Negative controls were included in each reaction as quality control. Moreover, 50% of randomly selected samples were genotyped twice, and the results were 100% concordant. All the analyses were performed in our non-accredited research laboratory at the Department of Pharmacy and Biotechnology, University of Bologna.

### 4.4. Statistical Analysis

Continuous variables are presented as mean ± standard deviation (SD), while categorical variables as absolute frequency. The association between cholesterol levels, response to statins, obesity, calculated as BMI or fat waist circumference, and the various genotypes were tested with Pearson’s chi-square test or analyses of variance as appropriate. The distribution of genotypes was tested for Hardy-Weinberg (HW) equilibrium. Multivariate logistic regression analyses were performed to evaluate the association between: genotypes and hypercholesterolemia and statin response (adjusted for age, sex, BMI or waist circumference), genotypes and obesity (adjusted for age and sex), genotypes and fat waist circumference (model by sex, adjusted for age). Results were presented as odd ratios (OR) and 95% confidence interval (95% CI). The level of significance was set at *p <* 0.05; Statistical analysis was conducted using Stata Intercooled version 12.0 [71].

## Figures and Tables

**Table 1:** Main demographic, clinical and laboratory characteristic of the Brisighella population—the hypercholesterolemic group included individuals with total cholesterol >200 mg/dL and subjects under therapy with statins.

Characteristics	All (*n.* 1622)	Hypercholesterolemia *	
		Yes (*n.* 965; 59.5%)	No (*n.* 657; 40.5%)
Age, years			
mean ± SD		58.6 ± 16.5	46.5 ± 17.3
Range		19–97	19–90
Gender *n* (%)			
Male	786 (48.5%)	456 (47.2%)	330 (50.2%)
Female	836 (51.5%)	509 (52.8%)	327 (49.8%)
Smoking Status ^†^			
Never smoker	903 (55.7%)	531 (55.0)	372 (56.6%)
Former smoker	403 (24.8%)	262 (27.1%)	141 (21.5%)
Current smoker	277 (17.1%)	150 (15.5%)	127 (19.3%)
BMI			
mean ± SD	26.5 ± 4.5	27.1 ± 4.4	25.8 ± 4.6
Range	16.5–47.5	16.5–46.1	17.1–47.5
Waist Circumference, cm			
mean ± SD	90.8 ± 12.9	92.8 ± 12.2	87.9 ± 13.3
Range	59–140	61–140	59–134
Total Cholesterol, mg/dL			
mean ± SD	204.0 ± 38.8	225 ± 33.4	172.2 ± 19.6
Range	96–354	111–354	96–199
LDL, mg/dL			
mean ± SD	135.6 ± 34.6	152.8 ± 32.1	110.3 ± 18.9
Range	35.6–264.2	39.6–264.2	35.6–149.4
HDL, mg/dL			
mean ± SD	46.0 ± 10.7	47.3 ±11.0	44.1 ± 10.0
Range	20–117	25–96	20–117
Triglycerides, mg/dL			
mean ± SD	112.8 ± 67.7	127.8 ± 69.4	90.8 ± 58.4
Range	11–809	11–546	11–809
APO B, mg/dL			
mean ± SD	86.3 ± 25.3	95.2 ± 24.7	73.8 ± 20.4
Range	0.7–189	0.7–189	0.8–150
APO A1, mg/dL			
mean ± SD	142.2 ± 29.0	145.4 ± 29.1	137.8 ± 28.3
Range	72–263	74–263	72–261

* Hypercholesterolemia: The group **YES** included individuals with total cholesterol >200 mg/dL and individual under therapy with statins; ^†^ smoking status is not available for 39 subjects.

**Table 2:** *KIF6* and *HMG-CoA* genotypes distribution in the studied population *.

Genotype	All (*n.* 1622)	Hypercholesterolemia	
		Yes (*n.* 965; 59.5%)	No (*n.* 657; 40.5%)
*KIF6*	*n*	*n*	*n*
rs20455			
AA	640 (39.6%)	402 (41.7%)	238 (36.4%)
AG	773 (47.8%)	444 (46.1%)	329 (50.3%)
GG	205 (12.6%)	118 (12.2%)	87 (13.3%)
Variant allele frequency (*q*)	*q =* 0.366	*q =* 0.353	*q =* 0.385
Hardy-Weinberg *p* value	*p* = 0.228	*p* = 0.787	*p* = 0.108
rs9471077			
GG	223 (13.8%)	129 (13.4%)	94 (14.3%)
GA	799 (49.3%)	460 (47.7%)	339 (51.8%)
AA	597 (36.9%)	375 (38.9%)	222 (33.9%)
Variant allele frequency (*q*)	*q =* 0.615	*q =* 0.628	*q =* 0.598
Hardy-Weinberg *p* value	*p* = 0.086	*p* = 0.518	*p* = 0.051
rs9462535			
AA	220 (13.6%)	129 (13.4%)	91 (13.9%)
AC	798 (49.3%)	457 (47.4%)	341 (52.1%)
CC	601 (37.1%)	378 (39.2%)	223 (34.0%)
Variant allele frequency (*q*)	*q =* 0.618	*q =* 0.629	*q =* 0.601
Hardy-Weinberg *p* value	*p* = 0.079	*p* = 0.621	*p* = 0.029
*HMG-CoA*			
rs3761740			
CC	1325 (81.8%)	788 (81.7%)	537 (82.0%)
CA	281 (17.4%)	170 (17.6%)	111 (16.9%)
AA	13 (0.8%)	6 (0.7%)	7 (1.1%)
Variant allele frequency (*q*)	*q =* 0.095	*q =* 0.094	*q =* 0.095
Hardy-Weinberg *p* value	*p* = 0.653	*p* = 0.329	*p* = 0.639
rs3846662			
AA	491 (30.3%)	281 (29.1%)	210 (32.1%)
AG	778 (48.1%)	456 (47.3%)	322 (49.2%)
GG	350 (21.6%)	228 (23.6%)	122 (18.7%)
Variant allele frequency (*q*)	*q =* 0.457	*q =* 0.473	*q =* 0.433
Hardy-Weinberg *p* value	*p* = 0.204	*p* = 0.106	*p* = 0.942

* More than 99% of the participants were successfully genotyped for all the variants.

**Table 3:** Influence of *KIF6* and *HMG-CoA* genotypes on hypercholesterolemia (TC >200 mg/dL) ^†^.

Genotype	Hypercholesterolemia			
	Yes (*n.* 965; 59.5%)	No (*n.* 657; 40.5%)	OR (95% CI)	*p* Value *
	*n* (%)	*n* (%)		
*KIF6*				
rs20455				
AA	402 (41.7%)	238 (36.4%)	1.0	
AG	444 (46.1%)	329 (50.3%)	0.84 (0.67–1.05)	0.127
GG	118 (12.2%)	87 (13.3%)	0.83 (0.59–1.17)	0.294
rs9471077				
GG	129 (%)	94 (%)	1.0	
GA	460 (%)	339 (%)	1.08 (0.78–1.49)	0.636
AA	375 (%)	222 (%)	1.29 (0.93–1.80)	0.133
rs9462535				
AA	129 (13.4%)	91 (13.9%)	1.0	
AC	457 (47.4%)	341 (52.1%)	1.02 (0.7–1.41)	0.776
CC	378 (39.2%)	223 (34.0%)	1.23 (0.88–1.71)	0.231
*HMG-CoA*				
rs3761740				
CC	788 (81.7%)	537 (82.0%)	1.0	
CA	170 (17.6%)	111 (16.9%)	1.04 (0.79–1.38)	0.776
AA	6 (0.70%)	7 (1.10%)	0.56 (0.18–1.77)	0.327
rs3846662				
AA	281 (29.1%)	210 (32.1%)	1.0	
AG	456 (47.3%)	322 (49.2%)	0.97 (0.76–1.2)	0.832
GG	228 (23.6%)	122 (18.7%)	1.40 (1.03–1.88)	0.030

* Age, Gender, and BMI adjusted; ^†^ More than 99% of the participants were successfully genotyped for all the variants.

**Table 4:** Influence of *KIF6* and *HMG-CoA* genotypes ^†^ on obesity, calculated according to BMI.

Genotype	Obese *			
	Yes (*n* = 328; 30.0%)	No (*n* = 1294; 70.0%)	OR (95% CI)	*p* Value ^‡^
*KIF6*	*n* (%)			
rs20455				
AA	144 (43.9%)	496 (38.5%)	1.0	
AG	148 (45.1%)	625 (48.4%)	0.83 (0.64–1.08)	0.174
GG	36 (11.0%)	169 (13.1%)	0.75 (0.50–1.13)	0.170
rs9471077				
GG	44 (13.4%)	179 (13.9%)	1.0	
GA	155 (47.3%)	644 (49.9%)	1.01 (0.70–1.48)	0.642
AA	129 (39.3%)	468 (36.3%)	1.14 (0.77–1.68)	0.510
rs9462535				
AA	42 (12.8%)	178 (13.8%)	1.0	
AC	155 (47.3%)	643 (49.8%)	1.06 (0.72–1.55)	0.777
CC	131 (39.9%)	470 (36.4%)	1.19 (0.80–1.76)	0.387
*HMG-CoA*				
rs3761740				
CC	270 (82.3%)	1055 (81.7%)	1.0	
CA	55 (16.8%)	226 (17.5%)	0.94 (0.68–1.30)	0.704
AA	3 (0.9%)	10 (0.8%)	1.17 (0.32–4.34)	0.809
rs3846662				
AA	106 (32.3%)	385 (29.8%)	1.0	
AG	154 (47.0%)	624 (48.4%)	0.84 (0.63–1.12)	0.231
GG	68 (20.7%)	282 (21.8%)	0.84 (0.60–1.19)	0.338

* Obese: BMI ≥30 kg/m^2^; ^‡^ Age and gender adjusted; ^†^ More than 99% of the participants were successfully genotyped for all the variants.

**Table 5:** Influence of *KIF6* and *HMG-CoA* genotypes ^†^ on obesity, calculated according to waist circumference in males.

Genotype	Obese *			
	Yes (*n.* 234; 30.0%)	No (*n.* 546; 70.0%)	OR (95% CI)	*p* Value ^‡^
*KIF6*	*n* (%)			
rs20455				
AA	113 (48.3%)	195 (35.9%)	1.0	
AG	92 (39.3%)	264 (48.5%)	0.61 (0.43–0.86)	0.004
GG	29 (12.4)	85 (15.6%)	0.58 (0.35–0.95)	0.029
rs9471077				
GG	32 (13.7%)	88 (16.2%)	1.0	
GA	97 (41.4%)	276 (50.7%)	1.03 (0.64–1.66)	0.897
AA	105 (44.9%)	180 (33.1%)	1.72 (1.06–2.79)	0.027
rs9462535				
AA	31 (13.2%)	86 (15.8%)	1.0	
AC	95 (40.6%)	274 (50.4%)	1.02 (0.63–1.66)	0.920
CC	108 (46.2%)	184 (33.8%)	1.72 (1.06–2.80)	0.027
*HMG-CoA*				
rs3761740				
CC	183 (78.2%)	431 (79.2%)	1.0	
CA	48 (20.5%)	106 (19.5%)	1.00 (0.68–1.48)	0.985
AA	3 (1.3%)	7 (1.3%)	0.98 (0.25–3.90)	0.975
rs3846662				
AA	73 (31.2%)	161 (29.6%)	1.0	
AG	114 (48.7%)	254 (46.7%)	0.90 (0.62–1.29)	0.553
GG	47 (20.1%)	129 (23.7%)	0.74 (0.48–1.16)	0.193

* Obese: waist circumference ≥100 cm; ^‡^ Age adjusted; ^†^ More than 99% of the participants were successfully genotyped for all the variants.

**Table 6:** Influence of *KIF6* and *HMG-CoA* genotypes ^†^ on obesity, calculated according to waist circumference in females.

Genotype	Obese *			
	Yes (*n.* 350; 42.6%)	No (*n.* 472; 57.4%)	OR (95% CI)	*p* Value ^‡^
*KIF6*	*n* (%)			
rs20455				
AA	140 (40.1%)	186 (39.5%)	1.0	
AG	172 (49.3%)	234 (49.7%)	1.05 (0.75–1.46)	0.791
GG	37 (10.6)	51 (10.8%)	1.14 (0.67–1.93)	0.630
rs9471077				
GG	46 (13.2%)	55 (11.7%)	1.0	
GA	174 (49.9%)	240 (50.9%)	0.89 (0.55–1.44)	0.630
AA	129 (36.9%)	177 (37.5%)	0.81 (0.49–1.34)	0.415
rs9462535				
AA	45 (12.9%)	56 (11.9%)	1.0	
AC	175 (50.1%)	242 (51.2%)	0.93 (0.57–1.50)	0.755
CC	129 (37.0%)	174 (36.9%)	0.85 (0.51–1.40)	0.526
*HMG-CoA*				
rs3761740				
CC	292 (83.7%)	401 (85.0%)	1.0	
CA	56 (16.0%)	70 (14.8%)	1.27 (0.82–1.95)	0.280
AA	1 (0.3%)	1 (0.2%)	1.28 (0.07–0.90)	0.869
rs3846662				
AA	114 (32.6%)	136 (28.9%)	1.0	
AG	168 (48.0%)	236 (50.1%)	0.73 (0.51–1.04)	0.083
GG	68 (19.4%)	99 (21.0%)	0.76 (0.49–1.18)	0.217

* Obese: waist circumference ≥90 cm; ^‡^ Age adjusted; ^†^ More than 99% of the participants were successfully genotyped for all the variants.

**Table 7:** Influence of *KIF6* and *HMG-CoA* genotypes ^†^ on hypercholesterolemia treatment response.

Genotype	Hypercholesterolemia Treatment Response			
	No (*n.* 122; 66.7%)	Yes (*n.* 61; 33.3%)	OR (95% CI)	*p* Value *
*KIF6*	*n* (%)			
rs20455				
AA	50 (41.3)	31 (50.8)	1.0	
AG	53 (43.8)	24 (39.4)	0.69 (0.35–1.36)	0.287
GG	18 (14.9)	6 (9.8)	0.54 (0.19–1.54)	0.250
rs9471077				
GG	23 (19.0)	6 (9.8)	1.0	
GA	53 (43.8)	25 (41.0)	1.70 (0.61–4.77)	0.310
AA	45 (37.2)	30 (49.2)	2.41 (0.87–6.69)	0.082
rs9462535				
AA	23 (19.0)	6 (9.8)	1.0	
AC	52 (43.0)	26 (42.6)	1.81 (0.65–5.06)	0.258
CC	46 (38.0)	29 (47.6)	2.34 (0.84–6.53)	0.103
*HMG-CoA*				
rs3761740				
CC	96 (79.4)	47 (77.1)	1.0	
CA	24 (19.8)	13 (21.3)	1.17 (0.54–2.54)	0.690
AA	1 (0.8)	1 (1.6)	1.76 (0.10–29.7)	0.695
rs3846662				
AA	35 (28.7)	19 (31.2)	1.0	
AG	66 (54.1)	31 (50.8)	0.90 (0.44–1.83)	0.769
GG	21 (17.2)	11 (18.0)	0.91 (0.36–2.31)	0.840

Responder NO: LDL ≥ 130 mg/dL; YES < 130 mg/dL. * Age, Gender, and waist circumference adjusted; ^†^ More than 99% of the participants were successfully genotyped for all the variants.

## Supplementary Materials

Supplementary materials can be found at www.mdpi.com/1422-0067/19/1/49/s1.
