# Supplementary material for: Relationship between Lipid Phenotypes, Overweight, Lipid Lowering Drug Response and KIF6 and HMG-CoA Genotypes in a Subset of the Brisighella Heart Study Population
Source: Int J Mol Sci. 2017 Dec 24;19(1):49. doi: 10.3390/ijms19010049 (PMC5795999; doi:10.3390/ijms19010049)
Supplement: Supplementary file 1 [file ijms-19-00049-s001.pdf]

Supplemental Table S1. ***KIF6*** **and** ***HMG-CoA*** **polymorphisms description.**

Unique rs Code	HGVS Nomenclature #	Protein Change	MAF/MAF *
KIF 6			
rs20455	NC_000006.12:g.39357302A>G	p.(Trp719Arg)	0.366/0.346
rs9471077	NC_000006.12:g.39340966A>G	Intronic region	0.615/0.636
rs9462535	NC_000006.12:g.39348016C>A	Intronic region	0.618/0.631
HMG-CoA			
rs3761740	NC_000005.10:g.75336308C>A	Near-gene 5′	0.095/0.079
rs3846662	NC_000005.10:g.75355259A>G	Intronic region	0.475/0.430

# HGVS nomenclature searched through Mutalyzer 2.0.26, released on 19 July 2017 (https://mutalyzer.nl/).

* MAF: Minor allele frequency - MAF/MAF: MAF in the overall Brisighella population/MAF in population from Toscana, in Italy.
